# Epigenetic Regulation of the Human Papillomavirus Life Cycle

**DOI:** 10.3390/pathogens9060483

**Published:** 2020-06-18

**Authors:** Michelle Mac, Cary A. Moody

**Affiliations:** 1 Department of Microbiology and Immunology, University of North Carolina at Chapel Hill, Chapel Hill, NC 27599, USA; mtmac@email.unc.edu; 2 Lineberger Comprehensive Cancer Center, University of North Carolina at Chapel Hill, Chapel Hill, NC 27599, USA

**Keywords:** HPV, life cycle, epigenetics, histone, DNA repair, DNA damage response

## Abstract

Persistent infection with certain types of human papillomaviruses (HPVs), termed high risk, presents a public health burden due to their association with multiple human cancers, including cervical cancer and an increasing number of head and neck cancers. Despite the development of prophylactic vaccines, the incidence of HPV-associated cancers remains high. In addition, no vaccine has yet been licensed for therapeutic use against pre-existing HPV infections and HPV-associated diseases. Although persistent HPV infection is the major risk factor for cancer development, additional genetic and epigenetic alterations are required for progression to the malignant phenotype. Unlike genetic mutations, the reversibility of epigenetic modifications makes epigenetic regulators ideal therapeutic targets for cancer therapy. This review article will highlight the recent advances in the understanding of epigenetic modifications associated with HPV infections, with a particular focus on the role of these epigenetic changes during different stages of the HPV life cycle that are closely associated with activation of DNA damage response pathways.

## 1. Introduction

Human papillomaviruses (HPVs) are small, non-enveloped, double-stranded DNA viruses that exhibit a strict tropism for cutaneous and mucosal (e.g., oropharynx, anogenital tract) epithelium [1]. More than 200 HPVs have been identified and sequenced [2]. Approximately one-third of all HPV types are classified as mucosal HPVs that specifically target the genital mucosa and can be categorized into high risk and low risk based on their oncogenicity [3]. The low-risk HPVs (e.g., HPV6 and 11) induce hyperproliferative lesions, often resulting in genital warts, but rarely progress into high-grade neoplasia and invasive malignant cancer. In contrast, there are approximately 12–15 high-risk genotypes (e.g., HPV16, 18, 31, and 45) that are etiological agents of cervical cancers [4,5,6], with 99% of cervical cancers containing high-risk HPV DNA and expressing the viral oncogenes E6 and E7 [4]. High-risk HPVs are also associated with the development of other anogenital malignancies such as penile, vulvar and anal carcinomas, as well as an increasing number of head and neck cancers, with oropharyngeal squamous cell carcinoma becoming the major HPV-associated cancer in recent years [7,8]. The incidence of HPV-associated oropharyngeal cancers constitutes up to 90% of all new cases of oropharyngeal cancers in the U.S. [8]. While the current FDA-licensed HPV vaccines appear to be highly efficacious in decreasing HPV-associated diseases, they are not therapeutic against pre-existing HPV infections or malignant progression [9].

## 2. The HPV Life Cycle

### 2.1. HPV Genome Structure

**Figure 1:**
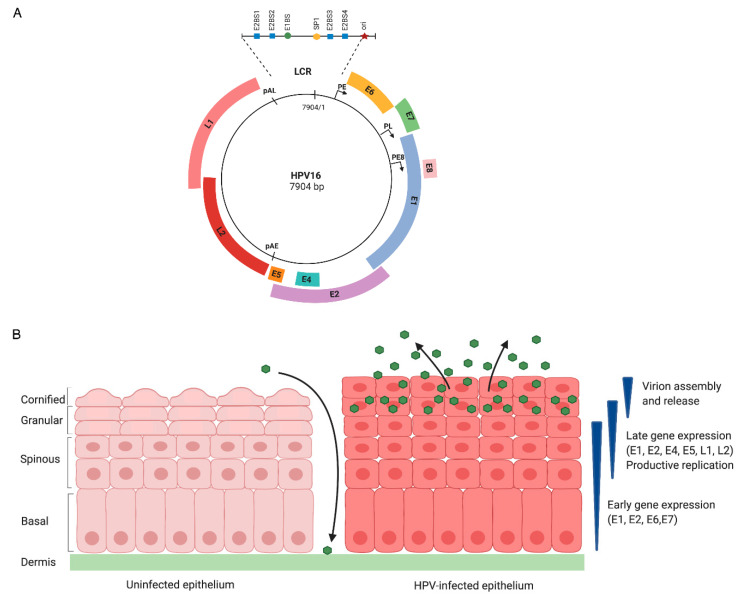
Genomic organization and the HPV life cycle. (**A**) The HPV genome consists of 6–8 open reading frames (ORFs) that are designated by the colored blocks. The early promoter (PE) is located upstream of the E6 ORF, and the late promoter (PL) is located in the E7 ORF. The early polyadenylation site (pAE) is located at the 3’ end of the E5 ORF, and the late polyadenylation site (pAL) is located at the end of the L1 ORF. The E8^E2 transcript is expressed from a promoter located in the E1 ORF (PE8). The Long Control Region (LCR) is an untranslated region that contains the keratinocyte enhancer (KE), origin of replication (ori), E1- (E1BS) and E2-binding sites (E2BS), as well as binding sites for various transcription factors. (**B**) Uninfected epithelium is shown on the left and HPV-infected epithelium is shown on the right. HPV infects the proliferating basal cells of the stratified epithelium exposed through a microwound. Upon entry, viral genomes are established in the nucleus of infected cells as episomes, early viral genes (E1, E2, E6, E7) are expressed, and the virus quickly amplifies to 50–100 copies per cell in an E1- and E2-dependent manner. HPV episomes are maintained at low-copy number in actively dividing basal keratinocytes by replicating along with cellular DNA. As infected cells divide, one cell remains in the basal layer, whereas the other cell migrates upward and initiates epithelial differentiation. Differentiation triggers the productive phase of the viral life cycle, resulting in viral genome amplification to thousands of copies per cell, late gene expression and virion assembly and release. The early promoter remains active, allowing for continued expression of E6 and E7 in differentiating cells. While differentiation normally results in an exit from the cell cycle, the E6 and E7 proteins deregulate cell cycle control to push differentiating cells back into the cell cycle, providing HPV access to cellular substrates required for productive viral replication. E4 and E5 also contribute to productive viral replication. Expression of L1 and L2 in the uppermost layers of the epithelium results in the assembly and release of virions.

In infected cells, the HPV genome exists as an extrachromosomal element (episome) of approximately 8 kilobase pairs that encodes for six to eight open reading frames (ORFs) (Figure 1A). Due to their limited coding capacity, HPVs support viral replication by manipulating host cell DNA replication and repair machinery [10]. The HPV life cycle is tightly associated with epithelial differentiation of host keratinocytes, in which the productive phase of the viral life cycle is restricted to the terminally differentiating suprabasal cells of the epithelium (Figure 1B). HPV infects the actively proliferating, undifferentiated basal keratinocytes of the stratified squamous epithelium that are thought to become exposed through a microlesion [11]. Two viral promoters, early and late, regulate viral gene expression and are active at different stages in the life cycle (Figure 1A) [12]. The early promoter (p97 for HPV16 and 31, p105 for HPV18) is located upstream of the E6 ORF and directs expression of early viral genes in undifferentiated cells, but remains active throughout differentiation (Figure 1A) [12]. The late promoter (p742 for HPV31, p670 for HPV16, p811 for HPV18) is located within E7 ORF and is activated upon epithelial differentiation to induce expression of late viral genes, including the L1 and L2 capsid genes [12]. The Long Control Region (LCR), also known as the Upstream Regulatory Region (URR), is an untranslated regulatory region that contains the keratinocyte enhancer (KE) region, origin of replication, and the early promoter. This region also contains binding sites for various transcription factors and the viral helicase E1 as well as the viral protein E2, which contributes to viral replication and regulation of viral gene expression [13]. 

### 2.2. The HPV Life Cycle Consists of Three Stages of Replication 

Following initial infection of exposed basal cells, the early promoter becomes active, resulting in expression of the E1 viral helicase, which along with E2, facilitates establishment replication, whereby viral episomes are quickly amplified to 50–100 copies per cell [14,15]. In these undifferentiated cells, viral genomes are subsequently maintained at low copy number by replicating along with cellular DNA. As the infected basal cells divide, viral DNA is partitioned to daughter cells—one of which migrates away from the basal layer and begins terminal differentiation. Epithelial differentiation induces the productive phase of the viral life cycle, leading to activation of the late promoter and expression of late viral genes (E4, E5, L1, L2), as well as high levels of E1 and E2 that drive viral genome amplification to thousands of copies per cell [12,16,17,18]. Virion assembly and release are restricted to uppermost layer of the epithelium as the immunogenic capsid proteins L1 and L2 are only expressed in highly differentiated suprabasal cells [19,20]. While normal epithelial cells exit the cell cycle upon differentiation, the E6 and E7 proteins deregulate normal cell cycle checkpoints to push differentiating cells back into the cell cycle, resulting in a G2 environment that provides cellular factors necessary for productive viral replication [10]. The E6 and E7 proteins deregulate cellular proliferation and apoptotic machinery in large part by targeting the tumor suppressor proteins p53 and pRb, respectively [21,22,23]. E7 promotes the degradation of pRb, leading to aberrant activity of E2F transcription factors that promote S phase re-entry of differentiating cells to provide a replication-competent environment [24,25]. Unscheduled cell cycle re-entry activates p53 to induce cell cycle arrest or apoptosis of infected cells; however, E6 circumvents these events by targeting p53 for degradation [26,27,28]. 

## 3. Epigenetic Regulation of HPV Gene Expression

### 3.1. Epigenetic Modifications of HPV Chromatin Regulate Viral Gene Expression Throughout the Viral Life Cycle

Epigenetics is defined as a post-translational modification process that affects gene expression but does not alter the underlying DNA sequence. In the virion and infected cells, HPV genomes are organized in the form of nucleosomes packaged into chromatin [29,30]. HPV genomes are epigenetically regulated by post-translational modifications of histones, including acetylation, phosphorylation and methylation, as well as by DNA methylation [31,32]. Histone modifications associated with distinct transcriptional states are controlled via a balance between histone readers such as histone acetyltransferase (HATs) and histone methyltransferases (KMTs) and histone erasers, including histone deacetylases (HDACs) and histone demethylases [33]. Regulation of viral gene expression in the different layers of the epithelium is critical to completion of the viral life cycle, and this control is achieved in large part through regulation of viral chromatin structure. 

The viral promoters are bound by nucleosomes in an ordered arrangement and are subjected to chromatin remodeling at different stages of the viral life cycle [34,35]. DNase I hypersensitivity analysis of HPV31 chromatin demonstrated a major chromatin rearrangement around the late promoter region that coincides with epithelial differentiation, marking readily accessible DNA regions for recruitment of transcriptional machinery [35]. Studies by Wooldridge et al. demonstrated that the early and late promoters of HPV31 exhibit a transcriptionally active chromatin configuration as indicated by the presence of dimethylated forms of H3K4 (H3K4me2) and acetylated H3 and H4 [34]. Upon differentiation, the levels of H3K4me2 and acetylated H3 around both promoter regions increase significantly. In addition, these histone modifications are associated with increased C/EBP-β binding to the KE/early promoter region and C/EBP-α binding to the late promoter upon differentiation [34], indicating a differentiation-dependent change in transcription factor binding to HPV promoter regions to regulate viral gene expression at different stages of the viral life cycle. Additionally, the C/EBP-β isoforms LAP and LIP have been shown to positively and negatively regulate expression of viral transcripts from the late promoter, respectively, upon differentiation in keratinocytes stably maintaining HPV31 episomes [36]. For HPV16 and HPV18, the KE/promoter region has been shown to be negatively regulated by YY1, which facilitates the recruitment of the polycomb repressor complexes 1 and 2 (PRC1 and PRC2) to viral chromatin [37,38,39,40,41,42]. PRC1 and PRC2 bind to the HPV18 LCR region and are associated with the enrichment of the repressive marks H3K27me3 and ubiquitinated H2AK199 (H2AK199Ub), respectively, leading to repression of early promoter activity and viral oncogene expression [42]. Interestingly, epithelial differentiation results in reduced YY1 expression and loss of epigenetic repression of the early promoter region, resulting in upregulation of HPV18 E6 and E7 expression [42]. These studies demonstrate that viral transcription is coordinated with histone modifications throughout the differentiation-dependent viral life cycle. 

### 3.2. Histone Acetylation 

Histone acetylation is a dynamic and reversible process that modulates gene expression by altering the spatial density of chromatin and is regulated by histone-modifying enzymes, including histone acetyltransferases (HATs) and histone deacetylases (HDACs) [43]. The activity of HATs and HDACs is tightly regulated to control the turnover of histone acetylation. Acetylation of lysine residues by HATs leads to transcriptionally active chromatin, whereas HDACs remove acetyl residues, thus marking transcriptionally repressed chromatin [43]. The HPV E6 and E7 oncoproteins promote a replication-competent environment in infected cells in part by modulating the expression and activities of HATs and HDACs [44]. The CREB-binding protein (CBP) and p300 are paralogous transcriptional coactivators with intrinsic HAT activity [45]. HPV16 E2 requires p300 to efficiently activate the early promoter and early gene expression [46]. Bernat et al. demonstrated using GST pull-down assays that high-risk HPV16 E7, and to a lesser extent, low-risk HPV11 E7 directly interact with p300 in vitro [47]. Using co-immunoprecipitation and mammalian two-hybrid assays, these authors also showed that HPV16 E7 interacts with p300 in vivo [48]. Additionally, they found that HPV16 E7’s interaction with p300 is necessary to inhibit p300’s ability to co-activate E2-driven transcription [47]. CBP mediates H3K14 acetylation and upregulates the transcriptional activity of the HPV18 URR [48]. HPV16 E7 associates with histone deacetylases HDAC1 and HDAC2 indirectly through Mi2β, a member of the nucleosome remodeling and histone deacetylation (NURD) complex [49,50], and this association is independent of Rb binding [50]. The binding of type I HDACs (HDACs 1, 2, 3) to HPV31 E7 directly modulates viral replication by activating E2F2-mediated transcription in suprabasal keratinocytes, which may promote S-phase re-entry of differentiating cells [21]. Additionally, mutation of the HPV31 E7 HDAC-binding domain in the context of the viral genome reduces episomal maintenance in undifferentiated cells and blocks productive replication upon differentiation [49]. Whether the E7–HDAC interaction impacts viral replication through modification of viral chromatin is currently unclear. HDAC1 and HDAC2 expression are also found to be elevated in cervical dysplasia and invasive carcinoma [51], suggesting that E7-dependent histone acetylation may be important for transcription regulation processes that drive HPV-induced tumorigenesis. 

Several HPVs express an E8^E2C protein, which functions as a transcriptional repressor from both promoter-proximal and distal E2-binding sites (E2BS) in the LCR and also negatively affects viral replication [52,53,54,55,56,57,58]. Ammermann et al. demonstrated using GST pull-down assays that HPV31 E8^E2C directly interacts with HDAC 1, 2, and 3 in vitro through the E8 domain, with inhibition of HDAC activity partially alleviating transcriptional repression mediated by the E8 domain [59]. In addition, proteomic analyses revealed an interaction between the E8 domain of the HPV31 and HPV16 E8^E2C proteins and the NCoR1/HDAC3 repressor complex, which is required for E8^E2C-mediated inhibition of transcription and replication [60]. The multi-subunit TIP60 histone acetyltransferase complex has also been implicated in the regulation of HPV gene expression. A genome-wide siRNA screen established a role for EP400, a component of the TIP60 complex, as well as the histone reader Brd4 in E2-mediated silencing of the HPV18 LCR and E6/E7 expression [61]. TIP60 and EP400 interact with HPV16 E2 and contribute to E2-mediated transcriptional silencing of the HPV18 URR, resulting in repression of E6 and E7 expression [61,62]. Whether E2’s interaction with TIP60 and EP400 is direct or indirect is currently unclear. TIP60 can also bind to the HPV18 early promoter in a YY1-dependent manner, resulting in histone acetylation that recruits Brd4 and represses E6/E7 expression [63]. E2 of multiple HPV types also directly interacts with Brd4 to negatively regulate viral gene expression [64].

### 3.3. DNA Methylation

DNA methylation is a post-replicative DNA modification that involves methylation of the 5’-position of cytosine residues located in CpG dinucleotides and is mediated by DNA methyltransferases (DNMTs) [65]. HPV DNA can be modulated by methylation, which affects viral gene transcription [66,67,68,69]. A key factor regulating transcriptional activity as well as replication of HPV genomes is the E2 protein. E2 regulates these viral processes by binding to E2-binding sites (E2BS) located within the LCR, which are partially palindromic sequences (5′-ACCGN_4_CGGT-3′) (Figure 1A) [70]. High-risk HPV genomes contain four highly conserved E2BS located near DNA-binding sites for several cellular transcription factors as well as the E1 viral helicase [70]. The binding of E2 to various E2BS activates or represses gene expression from the early promoter, as well as the recruitment of E1 to viral DNA [71]. Occupancy of the E2BS is controlled in part by the level of E2, with high levels of E2 binding to all E2BS, in turn repressing the early promoter as well as viral replication [71]. Additionally, methylation of CpG dinucleotides within the E2BSs is thought to inhibit E2 binding [66,67]. Upon differentiation, the three E2BS most proximal to the early promoter (E2BS2, 3, 4) become methylated, which may prevent E2-mediated repression of the early promoter during the productive phase of the life cycle [69]. Studies using HPV16-episome containing W12 cells, derived from a low-grade CIN1 cervical lesion, revealed that in contrast to the E2BSs, the early promoter region of the LCR becomes hypomethylated upon differentiation [66]. Together, these data indicate that the CpG methylation status of the viral DNA is dependent on the differentiation status of the cell, which likely influences viral gene expression over the course of the viral life cycle.

## 4. HPV-mediated Epigenetic Modifications of Cellular Chromatin

HPV infection can also induce modifications of host cellular chromatin, including DNA and histone methylation, resulting in aberrant expression of cellular genes. In high-risk HPV-associated lesions, epigenetic silencing of tumor suppressor genes such as p53 by DNA methylation in CpG island regions of gene promoters is often observed [72]. Disruption of CpG island methylation has been proposed as a potential biomarker for early detection or predicting the risk of cervical cancer precursors [73,74]. The activity of DNA methyltransferase 1 (DNMT1), the major methyltransferase responsible for maintaining DNA methylation patterns following cellular replication, is elevated in many tumors, including cervical cancers [65,75]. In addition, studies have shown that DNMT3A and 3B are highly upregulated in high-risk episomal HPV and HPV-positive cervical cancer cell lines, and many cellular genes are differentially methylated in cervical neoplasia [76,77]. Therefore, DNA methylation status may be a useful prognostic marker for early detection of cancer precursors. The HPV16 E6 and E7 oncoproteins regulate the expression and activity of DNMT1 through p53 degradation and direct binding, respectively, leading to aberrant DNA methylation of cellular and possibly viral DNA [78,79]. 

The histone methylation status of cellular chromatin can also be modified by HPV proteins, leading to aberrant cellular and viral gene expression. Studies by Smith et al. demonstrated that E2 recruits the H3K4 demethylase SMCX (JARID1C/KDM5C) to the HPV18 URR to transcriptionally repress early promoter activity, resulting in decreased E6/E7 expression [61,62]. In addition, HPV16 E7-expressing cells and tissue sections of HPV-positive squamous cervical carcinomas exhibit a reduction in global levels of the repressive mark H3K27me3, which is mediated by the upregulation of H3K27-specific KDM6A and KDM6B histones demethylases [80,81,82,83]. Interestingly, the formation of E2F6-containing polycomb repressor complexes, which bind to the H3K27me3 mark in order to transcriptionally repress chromatin, is also reduced in HPV16 E7-expressing cells [84]. Intriguingly, HPV-positive squamous cervical lesions exhibit reduced H3K27me3 despite increased expression of PRC2 methyltransferase EZH2 [81]. Furthermore, KDM6A epigenetically de-represses the cyclin-dependent kinase inhibitor p21, which is required for high-risk E7-expressing cells to survive E7-induced replication stress [85]. Together, these studies provide evidence that reduced levels of H3K27me3 in HPV-positive cells are mediated by combined activities of different histone-modifying enzymes. However, whether E7’s ability to induce KDM6A/KDM6B expression alters epigenetic marks on HPV chromatin is currently unclear. 

In addition, human foreskin keratinocytes (HFKs) expressing HPV16 E7 exhibit increased levels of activating marks such as H3K9 acetylation and H3K4 methylation, with both histone marks requiring E7’s Rb- and HDAC-binding domains [86]. Using ChIP analysis, Zhang et al. showed that HPV16 E7 increases histone acetylation on the promoters of E2F1 and cdc25a, two E2F-responsive genes that are increased in response to E7 expression [86]. The ability of E7 to increase histone acetylation may be necessary to active E2F-responsive genes that facilitate cell cycle re-entry, contributing to an extension of cellular life span, but also providing an environment conducive to viral replication in differentiating cells.

## 5. DNA Repair-Induced Epigenetic Modifications of HPV Chromatin

### 5.1. The DNA Damage Response

**Figure 2:**
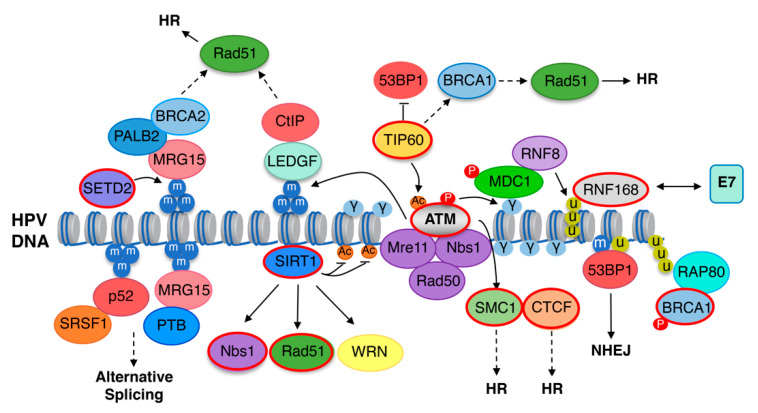
DNA repair-induced epigenetic regulation of the HPV viral life cycle. DDR components shown to play a role in the HPV life cycle are highlighted in red. Upon DNA damage, DSBs can be recognized by the MRN complex (MRE11/Rad50/Nbs1), which, together with TIP60 acetyltransferase, promotes the activation of ATM through phosphorylation (depicted as P) and acetylation (depicted as Ac), respectively. Activated ATM acts as a primary signal to induce a signaling cascade through phosphorylation of histone H2AX on Ser139, forming γH2AX at DNA breaks (depicted as γ). γH2AX promotes the recruitment of various DDR effectors in a highly regulated manner at sites of damage via the binding of scaffolding protein MDC1. MDC1 also recruits the E3 ubiquitin ligase ring finger 8 (RNF8) to initiate K63-linked ubiquitin chains (depicted as U) on the histone linker H1, leading to the recruitment of E3 ubiquitin RNF168. RNF168 specifically catalyzes ubiquitination of H2A/H2AX on lysine 13/15 (H2AK13/15ub): a modification essential for accumulation of 53BP1 as well as the RAP80–BRCA1 complex. High-risk HPV E7 proteins directly interact with RNF168. TIP60 has been shown to block 53BP1 recruitment, which may in turn block NHEJ (non-homologous end-joining) and promote HR repair through the recruitment of BRAC1 and Rad51 to viral chromatin. SIRT1 recruits Nbs1 and Rad51 to HPV chromatin. SCM1 is recruited to HPV genomes in association with CTCF—both of which may contribute to productive replication through recruitment of HR factors. SETD2 mediates trimethylation of histone H3K36 (H3K36me3, depicted as m) to recruit effector proteins to regulate multiple cellular processes, including HR (homologous recombination) repair and alternative splicing, which are processes critical to completion of the HPV life cycle. SETD2-mediated H3K36me3 may facilitate HR repair through the recruitment of LEDGF-CtIP and MRG15-PALB2-BRAC1 to viral chromatin. Additionally, H3K36me3 regulates alternative splicing through recruitment of p52-SRSF1 and MRG15-PTB. ATM activity is also required for H3K36me3 maintenance on viral chromatin through an unknown mechanism. Dashed lines represent links that have not been tested experimentally.

Studies over the past decade have established a critical role for the DNA damage response (DDR) in productive replication of high-risk HPV types (Figure 2) [15,87]. DDR pathways serve as an important mechanism for cellular survival by ensuring the fidelity of replication and maintenance of genomic stability [88]. In response to DNA damage, cells have evolved mechanisms to elicit various intrinsic DDR pathways that are mainly regulated by Ataxia-Telangiectasia-Mutated (ATM), ATM and Rad3-related (ATR) and DNA-dependent protein kinase (DNA-PK) kinases—all of which belong to the PI3-Kinase (PIK) family of kinases [88]. The presence of DNA double-strand breaks (DSBs) activates ATM and DNA-PKcs, which then facilitate repair through high-fidelity homologous recombination (HR) or error-prone non-homologous end joining (NHEJ), respectively [89]. Conversely, ATR is activated in response to single-stranded DNA generated upon replication stress as well as resection of DSBs [90]. ATM and ATR are constitutively active in high-risk HPV-infected cells, and inhibition of either ATM or ATR blocks productive viral replication [91,92,93,94]. Expression of high-risk E7 as well as the E1 helicase of low- and high-risk HPV types is sufficient to induce DNA damage and activation of the ATM and ATR DDR pathways [91,95,96,97]. While high-risk HPVs employ ATM and ATR components to ensure high fidelity of viral gene replication and amplification upon differentiation [15,87], it is currently unclear whether low-risk HPV types also require activation of these DDR pathways. 

Following induction of cellular DSBs, ATM is recruited to the break site and activated by the MRN complex (MRE11/Rad50/Nbs1) as well as acetylation by TIP60 [98]. The MRN complex also functions downstream of ATM activity to promote HR repair [88]. Upon activation, ATM phosphorylates the histone variant H2AX on Serine 139, referred to as γH2AX [99]. γH2AX regulates chromatin dynamics in response to DSBs by inducing the coordinated recruitment of DDR effectors through the binding of histone readers at sites of damage [100]. γH2AX is bound by the scaffolding protein MDC1 [101], which recruits the E3 ubiquitin ligase ring finger 8 (RNF8). RNF8 deposits polyubiquitin chains on the linker histone H1, in turn recruiting the E3 ubiquitin ligase RNF168 [102,103,104,105]. RNF168 specifically monoubiquitinates H2A/H2AX on lysine 13/15, which recruits the NHEJ promoting factor 53BP1 [102]. RNF168 also deposits K63-linked polyubiquitin chains that promote recruitment of the HR factor BRCA1 through the RAP80 ubiquitin-binding protein [102,106]. RNF168’s main function is to promote NHEJ repair through 53BP1 recruitment. However, RNF168 can also contribute to HR repair in S/G2 phases through the direct recruitment of the PALB2/BRCA2 complex to resected DSBs, which facilitates loading of the Rad51 recombinase [107]. In addition, 53BP1 has been shown to facilitate HR repair of heterochromatin-associated DSBs specifically in G2 by promoting ATM-dependent phosphorylation and inactivation of the heterochromatin building factor KAP1 [108].

### 5.2. The DNA Damage Response Promotes Productive Replication through Viral Chromatin Modifications

During the HPV life cycle, DDR activation is accompanied by significant alterations of viral chromatin that allow access of DNA repair proteins required for viral replication (Figure 2) [109]. γH2AX localizes to sites of HPV replication and is bound to viral chromatin, indicating a direct role for DNA repair factors in viral replication [110]. The DDR effectors 53BP1, Nbs1, BRCA1, and Rad51—all of which rely on γH2AX for recruitment to DNA breaks—also localize to HPV replication foci [110,111], supporting a role of γH2AX as an assembly center for the recruitment of repair factors to viral replication centers [109]. Indeed, Nbs1 and Mre11 of the MRN complex, along with the HR factors, Rad51 and BRCA1, are required for productive replication, indicating that HPV utilizes ATM activity to direct repair to HR on viral chromatin [112,113]. Additionally, a recent study by Sitz et al. demonstrated a critical role for RNF168 in the productive replication of HPV31 [114]. RNF168 protein levels are substantially upregulated in HPV31-positive cells, and transient depletion of RNF168 using small hairpin RNAs blocks productive viral replication upon differentiation, while having minimal effect on episomal maintenance in undifferentiated cells [114]. Importantly, this study also showed that high-risk, but not low-risk, E7 proteins directly interact with RNF168 via E7’s CR3 domain, hindering the function of RNF168 at cellular DSBs, resulting in decreased 53BP1 recruitment and an increase in HR repair [114]. Interestingly, recent studies from the Laimins lab demonstrated that breaks in the HPV31 genome are preferentially repaired at the expense of cellular DNA upon differentiation [115]. In unpublished studies, we have found that RNF168 and ubiquitin conjugates localize to large γH2AX domains that are used as surrogate markers of sites of productive replication (Huang and Moody, unpublished), indicating that RNF168 is active on viral chromatin. The localization of 53BP1 and BRCA1 to sites of productive HPV31 replication further supports RNF168 recruitment to viral chromatin and suggests E7 may titrate RNF168 away from cellular DSBs to direct host ubiquitin machinery to viral chromatin in response to ATM activity. RNF168 recruitment may, in turn, facilitate histone modifications that promote preferential recruitment of HR repair factors to allow for rapid repair of viral DNA during productive replication. Whether 53BP1 also plays a proviral role in productive replication is currently unclear. 

### 5.3. TIP60 Acetyltransferase and SIRT1 Deacetylase Are Required for Productive Viral Replication

Although TIP60 acetyltransferase has been implicated in negatively regulating HPV gene expression [61,62], TIP60 levels are increased in an E7- and STAT5-dependent manner in HPV31-positive cells and is required for viral replication [116]. TIP60 may contribute to productive replication through facilitating ATM activation. However, TIP60-dependent H4 acetylation at cellular DNA breaks promotes recruitment of HR factors by blocking 53BP1 chromatin association [117]. TIP60 may similarly modify viral chromatin to promote the recruitment of HR factors to sites of productive viral replication. SIRT1 deacetylase is also involved in chromatin modifications connected to the DDR [109,117,118,119]. In response to DNA damage, SIRT1 localizes to sites of DSBs and facilitates the recruitment of DNA repair factors to these regions. SIRT1 is increased in HPV31-positive cells in an E6- and E7-dependent manner and is required for episomal maintenance in undifferentiated cells as well as for productive replication and late gene expression upon differentiation [111,120]. SIRT1 depletion results in increased acetylation of H1K26 and H4K16 on HPV31 chromatin, corresponding with a block in Rad51 and Nbs1 recruitment to HPV31 DNA [111]. Whether SIRT1 modification of viral chromatin is required for the recruitment of Rad51 and Nbs1 to viral chromatin is unclear. However, SIRT1 modifies acetylation of non-histone proteins involved in DNA repair, including Nbs1 as well as the Werner DNA helicase, which regulates the fidelity of HPV16 E1-E2 replication in transient replication assays and may influence Rad51 and Nbs1 recruitment to viral DNA [121,122,123].

### 5.4. SMC1 and CTCF May Influence DNA Repair Factor Recruitment to Viral Chromatin

The Structural Maintenance of Chromosomes family protein SMC1 is phosphorylated by ATM in response to DNA damage and plays a central role in recruitment of HR repair factors to DSBs [124,125]. SMC1 is constitutively active in HPV31-positive cells and is required for productive replication [126]. In HPV31-positive cells, SMC1 is recruited to viral chromatin and forms a complex with the insulator transcription factor CTCF, which is important for DNA looping and three-dimensional chromatin interactions, but has also recently been shown to play a role in HR repair [127,128]. CTCF promotes HR repair through direct interaction with the CtIP endonuclease, which along with Mre11 mediates the initial end resection of DSBs required to initiate HR repair [129,130,131]. Depletion of CTCF or mutation of the conserved CTCF-binding sites in the L2 ORF of HPV31 blocks productive replication [126]. Whether SMC1 and/or CTCF contribute to productive replication through recruitment of HR factors to HPV chromatin is currently unclear. However, recent studies from the Parish lab demonstrated that CTCF epigenetically regulates the levels of high-risk E6 and E7 [42,132]. High-risk HPV types contain an additional conserved CTCF-binding site in the E2 ORF, with mutation of this site in the context of the HPV18 genome as well as CTCF depletion resulting in increased abundance of unspliced E6E7 transcripts and E6 and E7 protein levels [132]. The Parish group further showed that CTCF epigenetically represses E6/E7 expression through formation of a chromatin loop between the HPV18 E2 ORF and the LCR that is mediated through CTCF’s interaction with YY1 [42]. As mentioned, YY1 facilitates PRC1/2 recruitment and deposition of repressive H3K27me3 on the HPV18 LCR [42]. The decrease in YY1 upon differentiation disrupts the LCR-E2 ORF chromatin loop, leading to enhanced E6/E7 expression that supports productive viral replication [42]. These seminal studies were the first to identify a role for CTCF-dependent DNA looping in epigenetic regulation of the HPV life cycle, which may impact viral replication through regulation of E6/E7 gene expression, but also through recruitment of necessary HR repair factors to drive efficient viral DNA synthesis.

### 5.5. SETD2 and H3K36me3 Are Required for Productive Replication

Recent studies have shown that the HPV life cycle is also epigenetically regulated by SETD2 methyltransferase [133]. SETD2 interacts with the phosphorylated C-terminal domain of RNA polymerase II (RNAPII) and places the trimethyl mark on H3K36 (H3K36me3) during transcription elongation [134,135,136]. SETD2-mediated H3K36me3 recruits effector proteins to regulate multiple cellular processes, including alternative splicing as well as HR repair, mismatch repair, and the response to replication stress (Figure 2) [137,138,139,140,141]. SETD2 is elevated in high-risk HPV-positive cells through an E7-dependent increase in protein stability, and SETD2 depletion leads to defects in productive HPV31 replication as well as alternative splicing of late viral RNAs, specifically L1 [133]. SETD2 activity is required to maintain H3K36me3 on HPV31 chromatin, suggesting that SETD2 regulates the viral life cycle by recruiting H3K36me3 effectors to viral chromatin [133]. This is supported by the finding that overexpression of an H3K36me3 dominant negative mutant blocks productive replication [133]. Interestingly, ATM activity is also required for H3K36me3 maintenance on viral chromatin as well as splicing of late viral RNAs, identifying an additional role for ATM in the epigenetic regulation of the HPV life cycle [133]. While these findings support a significant role for SETD2-mediated H3K36me3 in epigenetic regulation of the HPV life cycle, the H3K36me3 readers that are recruited to viral chromatin have yet to be identified. However, H3K36me3 regulates multiple cellular processes that are critical for viral gene expression and replication, including HR factor recruitment through the binding of LEDGF, which in turn recruits CtIP and Rad51, as well as alterative splicing through recruitment of the Psip1 short isoform (p52) and MRG15, which in turn recruit the splicing factors SRSF1 and PTB, respectively (Figure 2) [137,140,141]. Interestingly, both SRSF1 and PTB have been shown to play a role in alternative splicing of HPV RNAs, with PTB facilitating splicing of L1 RNAs upon differentiation [142]. SETD2-mediated H3K36me3 may therefore regulate multiple aspects of the HPV life cycle.

### 5.6. Utilization of DDR Pathways during HPV Replication May Promote Viral Genome Integration and Genomic Instability

Even though high-risk HPVs utilize DDR pathways to facilitate viral replication, genomic instability is a hallmark of HPV-associated cancers, indicating that disruption of DNA damage repair mechanisms contribute to the initiation and progression of cancer [143]. HPV-induced genomic instability is driven by the expression of high-risk E6 and E7 oncoproteins, which induce replication stress and DNA damage, leading to the accumulation of chromosomal abnormalities [144,145]. In many HPV-associated cancers, the viral genome is found integrated into the host chromatin. Integration results in aberrant E6 and E7 expression that is thought to fuel cancer progression through increased genomic instability [146]. Viral genome integration is often found near common fragile sites [147], which are specific chromosomal regions susceptible to replication stress and chromosome breakage [148]. Interestingly, aberrant expression of E6 and E7 is in part controlled by epigenetic modifications at the HPV16 integrated loci such as the enrichment of activating marks H3K4me3 and H3K27ac [149,150]. HPV E2, in complex with cellular Brd4 proteins, is associated with viral replication foci that present adjacent to fragile regions [139]. This association conveniently provides access to DNA repair factoring for amplifying viral DNA; however, replication near fragile sites may increase the chance of viral integration, in turn driving carcinogenesis [151].

## 6. Summary and Outlook

Recent studies in HPV pathogenesis have revealed critical roles for epigenetic modifications in HPV infection and cervical carcinogenesis. Epigenetic modifications of HPV DNA are important for establishment replication, genome maintenance, and productive replication during the viral life cycle. It is clear that chromatin remodeling surrounding areas containing damaged DNA is required to allow access to DNA repair proteins that are necessary for HPV replication. Future investigation may focus on studying the specific histone marks that correlate with distinct DNA damage response pathways. As the incidence of HPV-associated oropharyngeal carcinoma continues to rise, expanding our knowledge of epigenetic drivers that regulate HPV pathogenesis in the oropharyngeal epithelium is an important area of future research. Even though epigenetic modifications in HPV-associated oropharyngeal cancers, including DNA methylation and histone modifications, have been reported [152], the role of the DNA damage response in the epigenetic regulation of the viral life cycle in oropharyngeal epithelium remains uncharacterized. This is especially important, as recent studies indicate that the viral genome is episomal in a large number of head and neck cancers [153]. Importantly, due to the reversible nature of epigenetic modifications, continued research may focus on investigating the role of epigenetic markers as biomarkers, their prognostic values, and therapeutic targets for HPV-associated cancers by using associated inhibitors. Further understanding of how epigenetic modifications regulate the HPV life cycle will provide insights for developing reliable targeted therapies for patients with HPV-associated lesions and cancers.
